# Animal Welfare, Agency, and Animal–Computer Interaction

**DOI:** 10.3390/ani15020219

**Published:** 2025-01-15

**Authors:** Heather Browning, Walter Veit

**Affiliations:** 1 Department of Philosophy, University of Southampton, Southampton SO17 1BF, UK; 2 Department of Philosophy, University of Reading, Reading RG6 6AA, UK; wrwveit@gmail.com

**Keywords:** animal welfare, agency, animal–computer interaction, choice, control, challenge

## Abstract

Recent discussions in animal welfare have emphasised the importance of animal agency—the ability of animals to make choices and exert control over their environment in a way that aligns with their needs and preferences. In this paper, we discuss the importance of animal agency for welfare and examine how use of some types of animal–computer interaction can enable animals to exercise more agency in captive environments through increased choice and control, cognitive challenge, and social interactions; as well as considering some of the potential limitations of such efforts.

## Simple Summary

Animal agency—the ability of animals to make choices and exert control over their environment in a way that aligns with their needs and preferences—is a key part of animal welfare. Animal–computer interactions can enhance animal agency and improve welfare through enabling choice and control over environmental conditions and social interactions and providing cognitive challenge. Though there are some potential limitations, with careful design and implementation, animal–computer interaction can be an important contributor to improving animal welfare.

## 1. Introduction

Animal agency refers to the ability of an animal to make choices and exert control over their environment in a way that aligns with their needs and preferences. Recent definitions of animal agency have emphasised animals’ motivation and capacity to freely engage with the environment. This means that animals are capable of forming basic goals (e.g., a desire for some resource) and recognising that their actions can influence whether they achieve them (e.g., receive the resource they desire). For instance, Littlewood et al. define agency as “the capacity of animals to engage in voluntary, self-generated, and goal-directed behavior that they are motivated to perform” [1] while Englund and Cronin define it as “the ability to successfully engage with the environment beyond satisfying immediate needs … by achieving goals, developing skills, acquiring information, and pursuing future plans” [2] and Špinka as “inner-motivated behavioural engagement with the environment” [3]. Agency is also closely related to the concepts of choice and control and in this paper, we will sometimes also talk about these functions. Choice refers to an animal having the ability to select between different options, whereas control refers to the ability to predictably influence aspects of the environment [2]. There is a growing recognition of the value of animal agency for animal welfare [1,2,3], and in this paper we will look at how this may be facilitated through use of technology, specifically animal–computer interaction (ACI).

There are several different competing definitions of animal welfare, with the most common being the “three orientations”: biological function, natural living, and affective state [4]. These can be taken as different aspects of welfare, or as independent theories of welfare. Here, we adopt the affective state (or hedonic) view, in which the welfare of an animal is made up of its positively and negatively valenced mental states, either at a time or over a lifetime (see [5] for a defence of this view). This is a commonly used definition within animal welfare science, forming the basis of the popular Five Domains model of animal welfare [6]. On this view, what matters for welfare is the range of affective states an animal experiences, both positive and negative. While sometimes simply referred to as “pleasures” and “pains”, in actuality these can include a wide range of different experiences, including bodily states such as hunger, nausea, and comfort; perceptual experiences such as pleasant or unpleasant sights, sounds, and smells; and responses to external conditions, such as fear or curiosity. On this definition of welfare, exercising agency will contribute to welfare when doing so creates positive welfare experiences. There are two ways in which agency may improve welfare. The first is directly, when the exercise of agency itself leads to positive affective experiences by the animal (e.g., joy, satisfaction). The second is indirectly, when animals use their agency to choose resources or activities they enjoy (e.g., selecting a favoured food resource). In this paper, we will look at both possibilities.

Regarding the direct welfare benefit of the exercise of agency, there is a growing body of evidence that animals find agency rewarding. For example, zoo animals given the choice between indoor and outdoor access will show lower incidence of stress-related behaviours, even if they only remain in one space [7]. Merely having the choice and being able to exercise agency over where they are located is enough to deliver a benefit. Other experiments have shown that primates and rodents, when given access to a switch that provides the option of turning a light in the enclosure on or off, will press it regardless of the initial light condition, again suggesting that the mere ability to control the environment is itself rewarding [8,9,10,11]. A recent review of choice-based animal welfare studies found a positive welfare benefit in most cases, although the study was unable to differentiate direct from indirect benefits [12]. There is also the observed phenomenon of *contrafreeloading* in which animals across a wide range of taxa will choose to work for a reward such as food, even when the same reward is also freely available [13,14]—for instance, one study found that maned wolves (*Chrysocyon brachyurus*) spent a lot of time searching for scattered food, even when food was simultaneously available on a tray [15]. Again, this suggests that the animals find it additionally rewarding when their actions lead to food access, as compared to just receiving the food on its own. While this evidence is suggestive, we also want to note that it is possible to interpret differently. Animals may pursue hard-wired instincts, rather than express genuine preferences. Some of the data for contrafreeloading may also just reflect boredom, rather than a desire for control. Nevertheless, sophisticated experiments control for alternative explanations and do not seem to be entirely reducible to them, thus leaving (at least some) room for the importance of agency. Indeed, it has been argued that agency has an adaptive benefit: the survival advantage of being motivated to make choices and perform flexible goal-directed behaviour, proximately motivated through accompanying positive affective states [3,16]. The direct importance of agency for welfare has been highlighted in a recent reworking of the Five Domains model, which has emphasised the agential nature of the fourth domain “Behavioural Interactions”, which they also term the “Agency Domain” [1].

On the indirect benefit, when animals exercise agency, they are able to make choices that can benefit their welfare. As we noted above, exercising agency is tied to an animal’s needs and preferences. Some very influential work in animal welfare science emphasises the importance of considering animal preferences [17,18]. Insofar as we take these to be linked to welfare (i.e., that what an animal needs and wants will typically be associated with valenced affective experiences), then animal agency can improve animal welfare by being better able to provide animals with welfare-enhancing conditions. Animal welfare science aims at discovering what conditions (e.g., food, shelter, social groups) are best for animals; but this is still a young science, and a difficult one. It is not always easy to determine what is best for an animal, and in many cases, it may be easier to allow them to decide for themselves (e.g., through providing choice of several shelters or substrates). This can also better account for differences among individuals, or changes in a single individual across time. However, this approach must always be taken with caution, as animals can choose according to their short-term desires while leading to their longer-term detriment (e.g., overconsumption of less healthy food options). Caregivers must also use their knowledge to appropriately constrain and guide animal choices when welfare is at risk, but this does not preclude the provision of choice wherever possible and practical.

As captive environments necessarily restrict the options and control available to captive animals, animal–computer interactions are one important way in which animals may be given increased agency in captive environments. Animal–computer interactions refer to a range of technological innovations that allow animals to directly interact with computers or similar digital technologies, not just responding to but also able to have input into the actions of computational devices, to perform actions such as choosing resources or activating environmental features [19,20]. These include screens, physical objects (e.g., buttons, toys), tracking technologies, and wearable technologies, among others [20]. In this paper, we will explore how ACI can enhance opportunities for animal agency and through doing so also improve animal welfare, in contexts such as farms, zoos, research laboratories, and domestic pets. It is not our intention here to provide a comprehensive review of the use of such technologies (see [20] for a thorough review), but rather to take some illustrative examples and examine them through the lens of how they might contribute to the growing interest in the role of animal agency for welfare improvements.

The rest of the paper will proceed as follows. In Section 2, we will look at how ACI promotes agency (and welfare) through enabling the animal to have choice and control over its environment. In Section 3, we will look at the potential social benefits of ACI. In Section 4, we will look at the benefits of ACI when used for cognitive challenge, such as through the provision of computer games. In Section 5, we will discuss some potential limitations of the use of ACI to enhance animal agency and welfare, and how they may be overcome. Finally, Section 6 concludes the discussion and points to some potential future research directions.

## 2. Environmental Choice and Control

The first, and possibly most important, role for ACI in enabling animal agency and enhancing welfare is through providing the animal with choices and control over their environment. As we have discussed in Section 1, this could have both direct benefits in terms of the positive feelings attendant with agency and indirect benefits in terms of allowing the animal to select what they want, when they want it, without needing to wait for the schedules of caregivers. Much of the work in this area has taken place in zoos, following the pioneering work on “behavioural engineering” by Hal Markowitz [21,22], who implemented a range of novel technological solutions for improving animal housing and husbandry, giving animals choice and control within their environments. However, even the basic “Skinner” boxes popular in laboratory animal research since the early 20th century, giving animals such as rats and pigeons control over lights, sounds, or food through interaction with buttons or levels [23], are a precursor to more complex forms of animal–computer interaction.

One way this can work is to give animals choices about their environmental conditions. Use of buttons or sensors can allow animals to choose, for example, the temperature and light levels within their environments. This allows them to solve their own problems (e.g., thermal discomfort) without the need for outside input from caregivers. They could even potentially be given choices over which habitats to occupy or trails to explore through use of RFID tags or similar devices [24]. Rhesus monkeys (*Macaca mulatta*), for instance, have been shown to be able to detect available choices [25,26,27,28]. Although this type of technology has typically been adopted within zoo settings [24,29], it could be at least as valuable in settings such as farms, where animals often have minimal choice over their conditions of living; however, there will obviously be limitations for animals living in large groups who may have differing preferences at any one time.

Animals can also be given options for environmental enrichments such as choices of ambient noise, or videos to watch. Although it is not uncommon for caregivers to provide auditory enrichments such as music or nature sounds [30], these are typically played directly into the enclosure without any say from the animals about what they want to hear, or whether they want any additional sound at all. Some research on provision of optional video or audio enrichment (in this case, saki monkeys [*Pithecia pithecia*] “opted in” by entering the tubes with the video or audio playback) showed that the animals would frequently choose to engage with these and would select noises that the experimenters had not expected them to like (e.g., traffic sounds) [31,32]. This last point is important, as it demonstrates the indirect value of giving the animals agency—animals may have preferences that surprise us and that we have not anticipated; and these preferences may change over time. Using ACI allows animals to choose when and what they experience in line with their own (potentially changing) desires.

A similar point holds for animals being able to activate various enrichment activities, such as showers or automated brushes [24]. Activities that rely on the presence of caregivers will be restricted by the (typically very busy) work schedules and may cause animals frustration when they have to wait for something they want. Many animals enjoy being sprayed with a hose, for instance, but have to wait for a caregiver to have time to provide them with this. Using ACI to allow animals to activate such activities (e.g., a sensor or button to switch on a shower) [24] allows them to choose when and for how long they participate, without being restricted by caregiver schedules. Access to automated brushes that do not rely on farmer presence have positively impacted cow welfare, with research showing they will work as hard to access these brushes as they will for fresh food [33]. Automated milking systems that allow cows to choose when they are milked can similarly increase cows’ agency [34]. Allowing animals to have the experiences they want or need at the time they want them can therefore have both direct and indirect welfare benefits.

This is especially important when considering the long hours most animals are left alone without caregivers present—overnight for farm, laboratory, and zoo animals and during the day for many domestic pets—when many activities are otherwise unavailable to them. Computer-activated feeding devices could even allow animals to choose when they have their food delivered, rather than waiting on caregiver schedules. Brando and Buchanan-Smith [35] have emphasised the importance of a “24/7” approach to animal welfare that provides for animals’ needs throughout the whole of their day, even when caregivers are not present. Particularly for nocturnal animals, overnight provisions may be more important than those during the day. ACI can allow animals to access what they need overnight, including ongoing feeding, even when caregivers are not present.

Finally, some forms of ACI may even allow animals to more directly communicate their needs through use of “interspecies communication devices”, such as lexigrams produced by chimpanzees (*Pan troglodytes*) (see [36] for a review). This is an especially important usage of ACI because it goes beyond the natural means animals typically have available to them to express their preferences and needs. Preference tests using touchscreens are the most obvious development in the literature and have shown promising results for practical applications across a range of species in laboratories and zoos [37,38,39]. Indeed, touchscreen tests may overcome some of the biases present in manual preference tests, such as the risk of cuing the animals to a particular option [37]. Preference tests have been implemented in species as diverse as American black bears (*Ursus americanus*) [40] and several species of turtles [41]. Auditory preference tests have also been conducted in gorillas (*Gorilla gorilla*) [42]. Other promising examples have been the use of “soundboards” with dogs [43]. These boards allow dogs to push a button to produce basic words (e.g., food, water, outside). Some dogs have been shown to understand a large number of words and can competently produce [44] and respond [45] to them appropriately. Although it has long been established that dogs can learn and understand a wide range of human words [46], until recently they have not had the capacity to produce words in return. These technologies provide a means by which animals can directly communicate to their human caregivers. Unlike some technologies that merely aim to interpret the usual vocal outputs of animals [47,48,49], these engage the agency of the animals—they can choose if and when to produce the words and choose what they want to say. While the research is still new and the results need to be replicated and confirmed, it seems undeniable that at the least the dogs are able to communicate some basic needs such as a desire for food, water, play, or to go outside. The ability to communicate one’s needs to another is a powerful agential tool, as it allows one to exert some control over the behaviour of others. Giving this tool to animals may have the direct benefits of having them feel this sense of agency within their interactions with caregivers, as well as the indirect benefits of receiving what they want or need when they want it, rather than when a caregiver makes the decision on their own. As humans are often busy and can miss many cues given by animals, direct communication circumvents some of the problems of human fallibility. It could also enhance agency within social interactions more generally, another benefit to which we will now turn.

## 3. Social Agency

A second similar role for ACI in enabling animal agency and welfare is through providing social choice and the attendant benefits. For many animals, interactions with conspecifics (or with keepers/handlers) is a central part of their welfare experience. The Five Domains framework has recently been updated to emphasise the importance of these types of behavioural interactions [6]. Using ACI to give animals more control over their social interactions could thus be another important way of improving their welfare, especially in laboratory settings where animals sometimes lack social contact

One example of this is the use of video technology to allow animals at different institutions or even different parts of the world to call and interact with one another. This has already been trialled with pet parrots. Parrots are social animals, and when kept alone they can suffer for the lack of companionship with conspecifics. The video calling technology allows the birds to select a social partner and place a call to them to view and communicate with one another over the video link [50]. The birds have agency over who and when they call, and although this study did not include the option, it would be relatively simple to allow birds to decide whether they want to receive a call. This allows them to make decisions about the social interactions they engage in. Other devices have allowed dogs to call their owners when they are away from the house [51]. Similar initiatives could be used for other species, particularly social species that have had to be kept alone for medical or management reasons. Work in laboratory mice has demonstrated a preference for watching videos of conspecifics engaging in social interactions [52], which may also have social welfare benefits, especially where animals are provided with choice over whether and when to watch such videos.

However, we would caution (as we will detail in Section 5), that this should not be seen as a replacement for the provision of appropriate social groups, but rather a remedial measure for improving welfare during periods of unavoidable isolation (e.g., when an animal is unwell, is awaiting a companion, or cannot be integrated into another social group), or an enhancement on top of normal forms of social interaction.

Another way in which ACI could be used to enhance animal welfare and agency along the social dimension is through providing methods that allow animals to choose their social companions. For example, use of sensor-activated doors (e.g., RFID chips, as mentioned in Section 2) can allow animals to choose when they enter an enclosure with conspecifics and when they spend time alone, without others being able to follow them. Females could choose if and when they have access to males in or out of breeding season. There is even the possibility of animals being able to select—say, through a touch-screen setup—which caregiver they would prefer for their daily care (though, of course, this will be restricted by limitations on staffing flexibility). As an anecdotal example, HB used to work with a solitary-housed chimpanzee who received her main social contact through time spent with keepers—having the option to signal to the keepers (e.g., through a button) when she wanted someone to visit her and who she wanted, may have helped her feel more in control of her social experiences. Some zoos have also employed technologies that allow animals to interact with visitors, such as competition between animals and visitors, or spraying them with jets of water or puffs of air [29]. Facilitating an increased range of social interactions and giving animals agency over how and when they occur is therefore an important potential welfare benefit of ACI.

## 4. Cognitive Challenge

A final role for animal–computer interaction is the provision of cognitive challenge, through computer games for animals. Although this may seem unusual, many of the welfare benefits animals derive from interactions with computers are likely to come in the form of satisfying a desire for curiosity and exploration (i.e., the SEEKING system; ref. [53]) that also provides them with cognitive stimulation. Indeed, in much of the research on choice and control via ACI, animals do seem to enjoy the exploratory aspects of exercising their agency, rather than via mere choice alone.

Notably, animal–computer interactions have been used in laboratory research (particularly for primates) for over 50 years [54,55], so it should not be surprising that these have developed to a sufficient level of complexity to earn the label “computer games for animals”. When voluntarily interacting with computer games, animals are exercising their agency in choosing how to spend their time. Indeed, some animals such as sun bears (*Helarctos malayanus*) show a preference for computerized enrichment over other forms of enrichment [56]. The cognitive challenges can also help in building competence in the form of cognitive and behavioural skills that can later be applied to other challenges and increase the range of behavioural options available to the animal, further enhancing agency [1,3,57,58]. This is similar to the role of play for young animals. Animal “computer games” could thus provide an ideal form of enrichment, enhancing welfare through challenge and the exercise of agency. Additionally, the use of cognitive games could not only enhance animal welfare but also tell us a lot about the cognitive abilities of animals in laboratory/research settings. Indeed, cognitive and psychological testing in laboratories has been one of the primary drivers of the initial development of animal–computer interaction technologies. We might see, for instance, animals surpassing new challenges previously assumed to be beyond their cognitive abilities, challenging preconceptions about animal intelligence.

Of course, this is not as simple as just providing human-oriented computer games to animals. Nonhuman animals have cognitive and physical differences from humans, including in many cases the lack of appropriate bodies to interact with computers. This then requires careful programming and design. Games designed for animals can be much simpler than the complex computer games available for humans, while nevertheless still counting as computer games. There are also a range of physical interface options that can be adapted for use by animals—even dolphins have been successfully provided with underwater keyboard systems [59]. Much ACI technology uses touchscreen interfaces, which have been used for a long time in zoos to test animals’ cognitive capacities as well as provide them with cognitive stimulation to improve their welfare [60]. As an example, Zoo Atlanta successfully implemented a touchscreen in a tree of the orangutan enclosure called the “Learning Tree” (https://zooatlanta.org/orangutan-learning-tree/, (accessed on 10 December 2024)). Here, they were able to run “games” such as the match-to-sample task, which involved matching a choice of pictures with a central one [61]. Success in these experiments resulted in automatically delivered food rewards. After this task, orangutans (*Pongo abelii*) were presented with a paint task that allowed them to select a colour to paint the screen without any food rewards [61]. Although in the first instance the status as a “game may be compromised by the extrinsic rewards, in the latter example the reward was the activity itself and is therefore arguably closer to a purer form of play. Related to the first role discussed in Section 2 (environmental control), touchscreens could also enable animals to choose between games to pick their favourite without any coercion—thus further enabling their agency.

Another computer game that was successfully implemented for animals is a digital version of Whack-a-Mole through the use of an underwater touchscreen in a dolphin enclosure at the National Aquarium Baltimore (https://www.rockefeller.edu/news/19742-researchers-create-interactive-touchscreen-dolphins/, (accessed on 10 December 2024)). Instead of a mole, however, the dolphins are able to interact with “swimming” fish on a screen. Overcoming the limitation of requiring physical touch, researchers have created acoustic “touchscreens” that operate via echolocation [62]. This means that animals less reliant on vision and touch, such as bats, may also be able to play similar games. Different animals have different internal models of the world (*umwelts*) arising from their varied perceptual capacities, and this will change how they experience their surroundings and interact with the environments they inhabit. All that is needed is that we open our imagination to new forms of cognitive games for animals very different from ourselves, with creative design options to accommodate their abilities.

This voluntary form of enrichment is a promising solution to the boredom and understimulation animals can face in captivity. Zoos are the perhaps perfect place to implement such enrichment devices, with much of the research taking place in collaboration between universities and zoos [63,64]. Lincoln Park Zoo, for instance, has used ACI to allow Snow Monkeys (*Macaca fuscata*) to select their offspring names, among other applications (https://www.lpzoo.org/pressroom/snow-monkey-uses-touch-screen-to-choose-name/, (accessed on 10 December 2024)). The American Society of Primatologists conducted interviews with primatologists on the potential usages of ACI for welfare and research, demonstrating the potential breadth of application within zoos and research laboratories (https://asp.org/2024/05/29/touchscreens/, (accessed on 10 December 2024)). Researchers have even used computers to give animals the opportunity to play games developed from behavioural economics [65]. Capuchins (*Cebus apella*), for instance, have been shown to fail to act prosocially in such economic games [66,67] while Western lowland gorillas (*Gorilla gorilla gorilla*) [68] succeeded. ACI research has also been conducted in less typically studied species, such as researching image discriminations in American black bears (*Ursus americanus*) [69]. Similar cognitive challenges could be introduced in other contexts such as farms or laboratories, where animals typically have fewer enrichment options than in zoos. These could be additionally valuable, especially for research into cognitive capacities and cognitive enrichment.

A further benefit of computer games for animals might be the fostering of cooperation. While touchscreen tests are usually restricted to one animal, it is in principle conceivable that we could implement so-called multiplayer games for animals that would require them to cooperate with one another or perhaps a human (e.g., a caregiver or visitor). This is linked to the potential social benefits of ACI, as discussed in Section 3.

Additional welfare benefits from computer games could come in the form of physical exercise. Although computer games in humans are often associated with a lack of movement, ACI setups can be designed to encourage more movement. For instance, small animals may chase a moving point on a screen that is comparatively large for their size, requiring them to move around. Indeed, touchscreen studies in laboratory mice and rats are now quite common, perhaps unsurprisingly due to their status as model organisms in research [70,71,72,73,74,75,76]. Motion-sensor technology, as has been used with orangutans [77], is another way of moving ACI beyond a single screen and encouraging more widespread movement. Physical exercise is another means of developing competence for future agency, as described above.

## 5. Possible Problems and Limitations

From our discussion throughout this paper, it should be clear that ACI holds many potential benefits for animals in terms of enabling agency through choice and control over environmental conditions and social interactions, as well as providing cognitive challenges. This of course does not mean that there cannot be any problems with it or limitations to its implementation. Nevertheless, we shall argue in this section that arguments against the use of ACI do not succeed; and that careful design and ongoing research can overcome many of the limitations.

Perhaps the strongest objection to the use of ACI is that it is bad for animals, in the sense of making their lives less natural. This comes from one of the common definitions of welfare as consisting in the living of a natural life in accordance with the evolved “telos” of the species [78]. We think this is unwarranted, as simulated environments can bring many of the benefits of real environments [79,80] and therefore constitute a great opportunity for increasing animal agency and welfare. Furthermore, as we alluded to earlier in this paper, we do not think that the idea of naturalness being a part of welfare, while popular, has merit. There has been a lot of work aiming to show why naturalness should not be considered an intrinsic component of welfare, but should rather be considered instrumentally valuable [5,81,82]. Concerns for naturalness likely arose as a response to animals housed in appallingly impoverished conditions—such that the wild seemed idyllic in comparison—but this does not have to mean that a completely natural life is the best life for an animal. Animals housed in captivity can benefit from many of the “unnatural” interventions provided, such as treatment of diseases and parasites, shelter from climatic extremes, and ongoing access to food and water sources. Focussing on the affective states the animal experiences shows that neither captivity nor a “natural” life in the wild are inherently better; what matters is the provisions for the animals’ needs. Thinking about zoos: while animal enclosures were historically quite barren and small, significant efforts have been made to provide animals with various forms of enrichment to keep them engaged. It is even possible that animals in captivity could have a more enriched life than most wild animals if sufficient effort is made to provide the necessary resources and opportunities. Though it is, of course, true that many captive environments are both unnatural and impoverished, we take the latter to be the primary problem and ACI to be one of the solutions.

A related problem to concerns about naturalness is that ACI technologies may be unpopular with visitors or the general public. Particularly in zoos, there is often a worry about using unnatural forms of enrichment where visitors can see [83]. It is well established that naturalistic enclosure design is important to zoo visitors [84,85,86,87,88,89], and this then raises the concern that unnatural additions could reduce the sense of immersion and the idea that an enclosure is representative of the environments these animals may have been surrounded with in the wild. However, such worries appear to be misguided. Researchers have found that zoo visitor attitudes towards the use of a touchscreen embedded in a tree were actually highly positive, evaluating it as both acceptable and likely to improve animal welfare [61]. Perhaps large, overt unnatural objects may create a more negative response, but this can easily be accommodated by trying to implement ACI in as naturalistic a manner as possible. Again, careful design that takes into account both the needs of the animal and of the institution is key.

In line with this is the fact that ACI is designed by humans and can thus have inherent biases toward human, rather than animal use. Several researchers have raised concerns that as animals are not able to participate in the design of ACI technologies, these could become exploitative [64,90,91]. Even when they are designed with the aim to improve animal agency and welfare, they may still only reflect what humans think is best for the animals rather than what the animals themselves want or need [64]. Yet, though we think this concern is worth taking seriously, we believe that these worries are overblown. Discussions of ACI are already taking very seriously the importance of considering the perspective of animals as key stakeholders [19,92]. For instance, Webber et al. [57] are directly responding to this worry in their call for “animal-centric technology design” that takes the animal’s point of view. While we have to be careful when considering the animals’ point of view, it is not true that we cannot get any input from animals regarding the design of ACI. Non-linguistic reports can still be valuable, and there are methods developed from animal welfare scientists using interpretation of animal behaviour to better understand animals’ interests. It is therefore entirely possible for ACI designers to pay attention to animals in the design and refinement of prototypes for ACI technologies. Additionally, this technology can be useful for gathering data about animals. This is valuable both for humans (in terms of increasing knowledge) and animals (when the knowledge gained can be applied to improved husbandry and welfare), and ideal design should facilitate this.

Fundamentally, there is no difference between ACI and other technologies in regard to this risk. There is no real alternative to taking a human perspective of trying to understand what is best for the animals; though ACIs themselves may help provide part of the picture, in an iterative process. What matters is to ensure we do not just assume what animals’ interests are without investigating but put careful time and effort into understanding the animal end users. There needs to be a prime directive for ACI, that it should be fundamentally designed with animal welfare in mind such that this technology is primarily for the animals themselves. This also needs to take into account the differences between individuals, not just species. However, through providing choices, well-designed ACI can better account for individual differences than many other traditional methods of provision.

Another potential problem is that ACI could be used in place of other more important welfare provisions. We raised this concern in Section 3, when discussing the social side of ACI. If ACI was used to provide animals with digital playmates instead of real conspecifics, this could be an overall harm to the animals. The same is true for replacing other “real” activities, such as providing computer games in place of physical or behavioural challenges. This is why it is important for ACI to be treated as an addition to, rather than a replacement for, other important factors that can improve animal welfare. This can also help prevent potential behavioural, cognitive, and developmental problems that might arise from an overuse of computer interactions. Just as with children, one may limit an animal’s screen time following research to determine what is safe or optimal.

Time limitations would also help allay concerns that animals could end up forced to “play” computer games or engage in ACI more generally in order to receive access to food or other valuable resources. It would be a bad outcome if animals were to be trapped in the equivalent of Skinnerian operant conditioning chambers in which they have to perform a desired task to receive food. This would end up doing the opposite of what is intended, reducing the choices available to the animals and their opportunities for agency. However, we do not see this as an issue—many ACI initiatives in zoos have shown animals will engage with touchscreens or other technologies even when food is not withheld. Indeed, we mentioned the phenomenon of contrafreeloading in Section 1, where many animals seem to prefer working for food rewards rather than getting food for free [13,14], and the same has been observed for ACI [61]. So long as the animals have both options, there is not a problem. Again, it should be a supplement to rather than a replacement for other means of acquisition.

On the opposite end are the worries that ACI may be too desirable and where typical interfaces can only be used by one animal at a time, this could lead to agonistic behaviours and worsen social relations in groups [61]. This was observed to some extent in orangutans (*Pongo pygmaeus*), who showed aggression around ACI, leading researchers to recommend these tools as perhaps best suited for solitary animals [93]. Nevertheless, some of the same researchers noted in a different paper that these challenges can likely be overcome if there are sufficient alternative enrichment opportunities “in the environment (e.g., foraging, social interaction, watching the public, etc.)” ([61], p. 29). Other forms of careful implementation could offset this effect, such as having multiple devices available, or setting limits on how long different individuals can use devices before they are locked out (using individual recognition). Again, it is crucial to think about the typical behaviour of the animals in the design phase and use prototype testing and refinement to identify and overcome any such issues if they arise.

The final set of problems refer to the pragmatic constraints on implementing ACI on-site. First, the technologies are likely to be expensive, and many institutions housing animals lack funds for such extras. A survey of zoo professionals indicated cost to be the primary barrier to implementing such technologies [29]. For contexts such as farming, introducing costly technologies could raise prices beyond what consumers are willing to pay, making them non-viable. The added financial pressures of using ACI could increase stress on institutions who feel like they need to introduce these to meet their ethical duties to their animals, or price them out of the market. However, we think these are manageable problems. ACI technologies can range from simple to complex, and not all institutions need to implement all of them. Rather, they can focus on what is affordable for their context. Zoos already invest in environmental design and enrichment, and as we believe welfare should be the ultimate aim of zoos [89], this investment is worthwhile. Research has also shown that visitors pay attention to animal welfare [94], and this also therefore makes good business sense. It is also important to note that ACI is not the only option available, and animals can also be provided with other opportunities to engage their agency, choice, and control. It is valuable, but not necessary, for improving welfare.

Second, it can be difficult to design ACI in a form that allows the animal to safely interact with it, without breaking the device and/or putting themselves at risk. Many animals are large and strong and can easily break objects designed for humans, such as tablets. This requires careful design and prototype testing before allowing animals unsupervised access, further adding to expense. The requirement for specialised staff to maintain and repair damaged equipment can also be costly [29]. This, therefore, largely falls under the financial concerns already discussed as well as provides further emphasis on the need for careful design and prototype testing with an eye for the capacities and needs of the animals [57].

Overall, ACI has some problems and limitations that need to be considered in contrast to the hype surrounding these technologies. But as we have shown here, many of these can be overcome, and those involved with animals should have an open mind about how ACI can be used to enhance animal agency and welfare.

## 6. Conclusions

We hope to have demonstrated in this paper that animal–computer interaction (ACI) bears great potential to improve the welfare of animals by enhancing their agency as well as to offer us opportunities to learn about their cognitive abilities. We have discussed three of what we consider the most promising pathways.

First, ACI can provide animals with control over their environments as well as the opportunity to engage in more choices. Typically, animals in captivity have limited control over their environments, which can cause frustration and boredom, compared to the environments of wild animals. Here, ACI could even go beyond these environments by giving animals the opportunity to engage in more choices and have more control than is possible for their wild counterparts. Further, as caregivers necessarily spend only limited time with their animals, ACI can provide animals with round-the-clock enrichment opportunities they would otherwise lack.

Second, we have discussed the benefits of ACI in enhancing animals’ social agency. Although use of such technology might seem solitary at first, it has already been applied in several contexts to enable communication between animals across different sites as well as communication between animals and their caregivers.

Finally, we discussed the possibility of computer games for animals as a form of cognitive stimulation. What may initially sound absurd has in fact already been a wide success. Many of the computerised tasks developed to probe the cognitive abilities of other animals can already be considered as legitimate games, such as whack-a-mole or matching games. These games can provide animals with an opportunity to exercise their agency and cognitive control to improve their welfare and develop competence to face future challenges, among other benefits such as the potential for physical exercise.

While there are potential problems and limitations to ACI, we have argued that many of these have been exaggerated and can be overcome. Particularly for novel applications in zoos, farms, and companion animals, paying attention to the longer history of ACI in laboratory research (e.g., through use of computer game tasks) could help solve problems of design and implementation. For a technology that is still in its infancy, there is much promise here to improve animal welfare by allowing animals to exercise their agency across a range of domains. Ongoing research and development in ACI should bear this in mind, maintaining a focus on centering the animals as agents and how they can benefit from using the technology.

## Data Availability

No new data were created or analyzed in this study. Data sharing is not applicable to this article.
